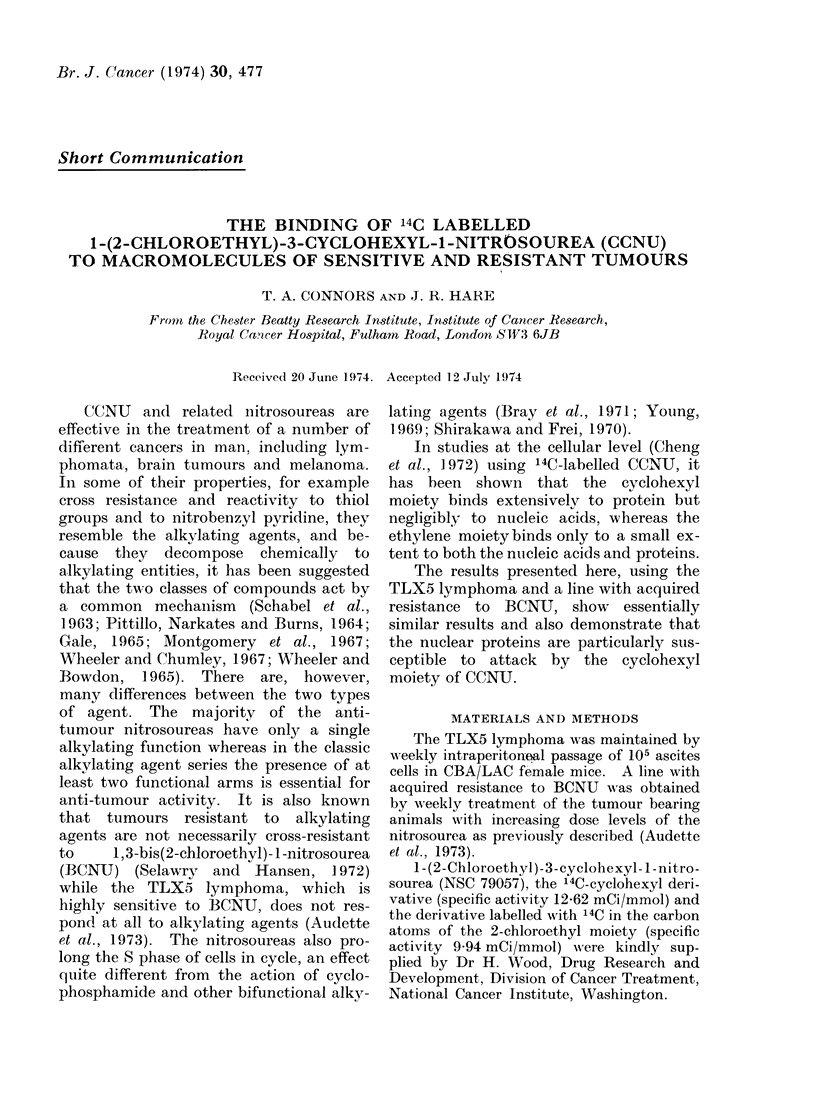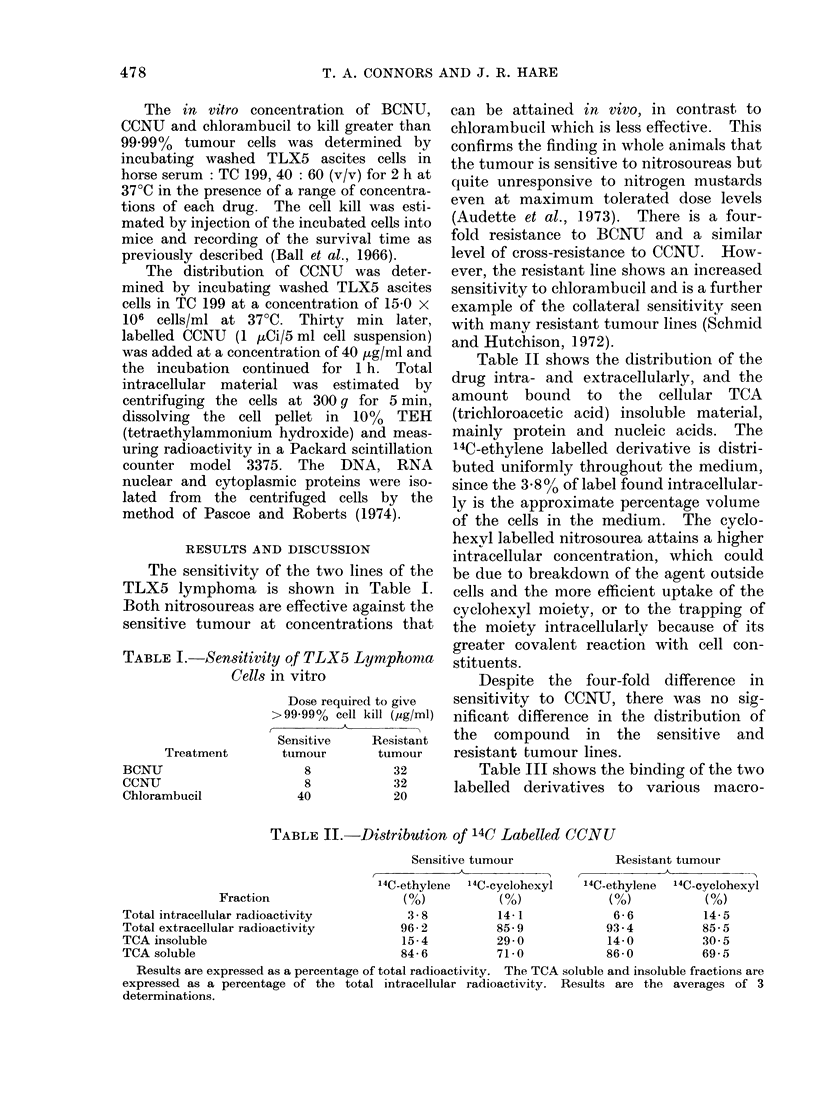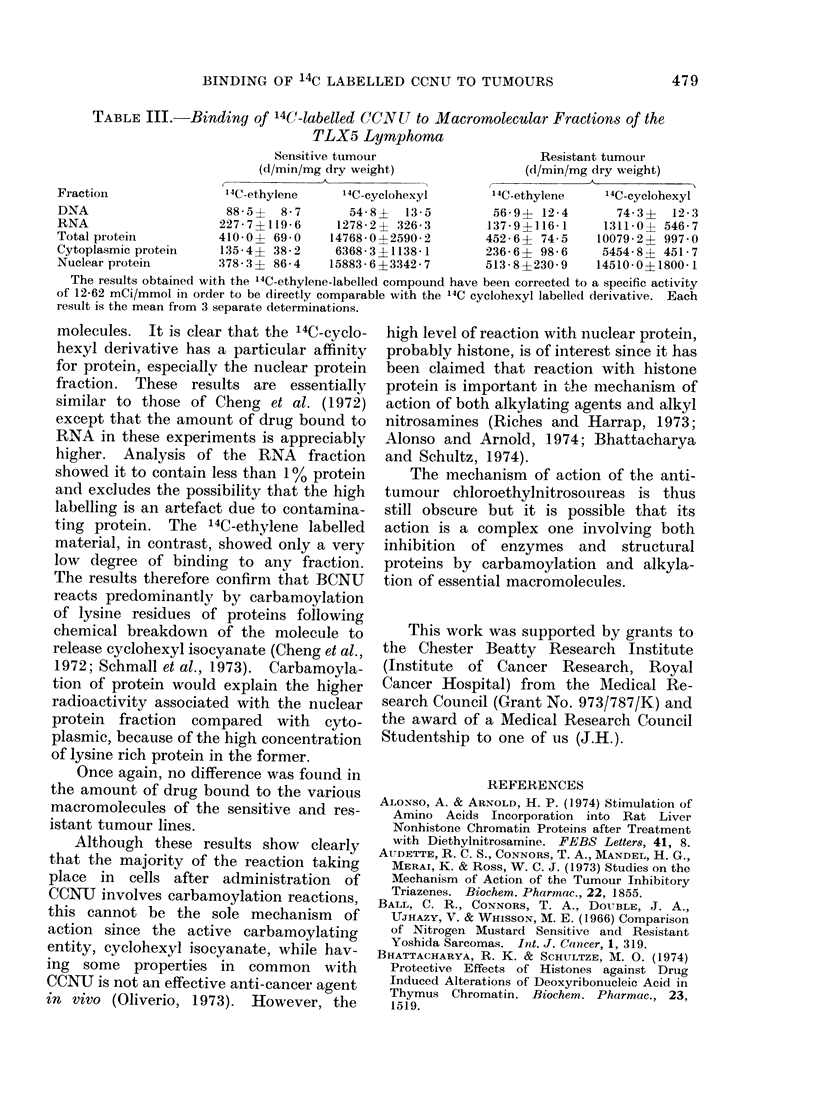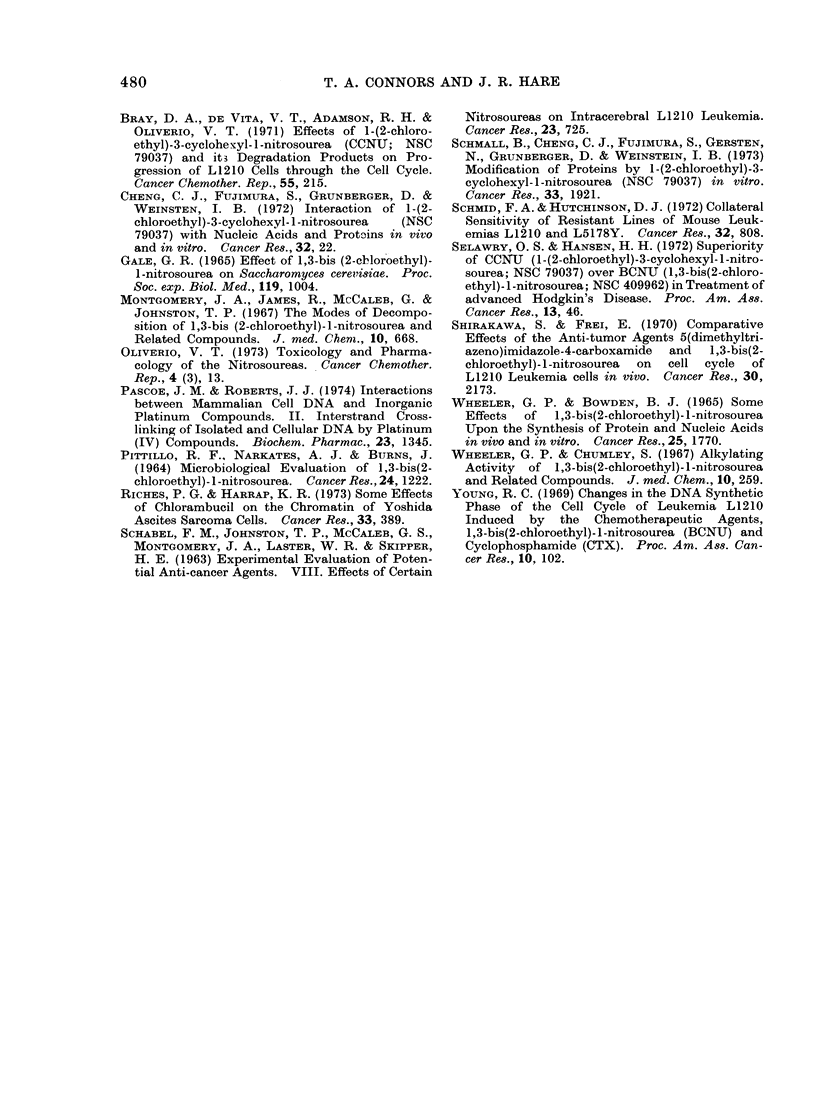# The binding of 14C labelled 1-(2-chloroethyl)-3-cyclohexyl-1-nitrosourea (CCNU) to macromolecules of sensitive and resistant tumours.

**DOI:** 10.1038/bjc.1974.224

**Published:** 1974-11

**Authors:** T. A. Connors, J. R. Hare


*Br. J. Cancer* (1974) **30**, 477

***Short Communication***

### THE BINDING OF $^{14}C$ LABELLED 1-(2-CHLOROETHYL)-3-CYCLOHEXYL-1-NITROSOUREA (CCNU) TO MACROMOLECULES OF SENSITIVE AND RESISTANT TUMOURS

T. A. CONNORS AND J. R. HARE

*From the Chester Beatty Research Institute, Institute of Cancer Research, Royal Cancer Hospital, Fulham Road, London SW3 6JB*

Received 20 June 1974. Accepted 12 July 1974

CCNU and related nitrosoureas are effective in the treatment of a number of different cancers in man, including lymphomata, brain tumours and melanoma. In some of their properties, for example cross resistance and reactivity to thiol groups and to nitrobenzyl pyridine, they resemble the alkylating agents, and because they decompose chemically to alkylating entities, it has been suggested that the two classes of compounds act by a common mechanism (Schabel *et al.*, 1963; Pittillo, Narkates and Burns, 1964; Gale, 1965; Montgomery *et al.*, 1967; Wheeler and Chumley, 1967; Wheeler and Bowdon, 1965). There are, however, many differences between the two types of agent. The majority of the anti-tumour nitrosoureas have only a single alkylating function whereas in the classic alkylating agent series the presence of at least two functional arms is essential for anti-tumour activity. It is also known that tumours resistant to alkylating agents are not necessarily cross-resistant to 1,3-bis(2-chloroethyl)-1-nitrosourea (BCNU) (Selawry and Hansen, 1972) while the TLX5 lymphoma, which is highly sensitive to BCNU, does not respond at all to alkylating agents (Audette *et al.*, 1973). The nitrosoureas also prolong the S phase of cells in cycle, an effect quite different from the action of cyclophosphamide and other bifunctional alkylating agents (Bray *et al.*, 1971; Young, 1969; Shirakawa and Frei, 1970).

In studies at the cellular level (Cheng *et al.*, 1972) using $^{14}C$-labelled CCNU, it has been shown that the cyclohexyl moiety binds extensively to protein but negligibly to nucleic acids, whereas the ethylene moiety binds only to a small extent to both the nucleic acids and proteins.

The results presented here, using the TLX5 lymphoma and a line with acquired resistance to BCNU, show essentially similar results and also demonstrate that the nuclear proteins are particularly susceptible to attack by the cyclohexyl moiety of CCNU.

### MATERIALS AND METHODS

The TLX5 lymphoma was maintained by weekly intraperitoneal passage of $10^5$ ascites cells in CBA/LAC female mice. A line with acquired resistance to BCNU was obtained by weekly treatment of the tumour bearing animals with increasing dose levels of the nitrosourea as previously described (Audette *et al.*, 1973).

1-(2-Chloroethyl)-3-cyclohexyl-1-nitrosourea (NSC 79057), the $^{14}C$-cyclohexyl derivative (specific activity 12·62 mCi/mmol) and the derivative labelled with $^{14}C$ in the carbon atoms of the 2-chloroethyl moiety (specific activity 9·94 mCi/mmol) were kindly supplied by Dr H. Wood, Drug Research and Development, Division of Cancer Treatment, National Cancer Institute, Washington.

The *in vitro* concentration of BCNU, CCNU and chlorambucil to kill greater than 99·99% tumour cells was determined by incubating washed TLX5 ascites cells in horse serum : TC 199, 40 : 60 (v/v) for 2 h at 37°C in the presence of a range of concentrations of each drug. The cell kill was estimated by injection of the incubated cells into mice and recording of the survival time as previously described (Ball *et al.*, 1966).

The distribution of CCNU was determined by incubating washed TLX5 ascites cells in TC 199 at a concentration of $15 \cdot 0 \times 10^6$ cells/ml at 37°C. Thirty min later, labelled CCNU (1 $\mu$Ci/5 ml cell suspension) was added at a concentration of 40 $\mu$g/ml and the incubation continued for 1 h. Total intracellular material was estimated by centrifuging the cells at 300 *g* for 5 min, dissolving the cell pellet in 10% TEH (tetraethylammonium hydroxide) and measuring radioactivity in a Packard scintillation counter model 3375. The DNA, RNA nuclear and cytoplasmic proteins were isolated from the centrifuged cells by the method of Pascoe and Roberts (1974).

### RESULTS AND DISCUSSION

The sensitivity of the two lines of the TLX5 lymphoma is shown in Table I. Both nitrosoureas are effective against the sensitive tumour at concentrations that can be attained *in vivo*, in contrast to chlorambucil which is less effective. This confirms the finding in whole animals that the tumour is sensitive to nitrosoureas but quite unresponsive to nitrogen mustards even at maximum tolerated dose levels (Audette *et al.*, 1973). There is a four-fold resistance to BCNU and a similar level of cross-resistance to CCNU. However, the resistant line shows an increased sensitivity to chlorambucil and is a further example of the collateral sensitivity seen with many resistant tumour lines (Schmid and Hutchison, 1972).

TABLE I.—*Sensitivity of TLX5 Lymphoma Cells* in vitro

| Treatment | Dose required to give >99·99% cell kill ($\mu$g/ml) Sensitive tumour | Resistant tumour |
|---|---|---|
| BCNU | 8 | 32 |
| CCNU | 8 | 32 |
| Chlorambucil | 40 | 20 |

Table II shows the distribution of the drug intra- and extracellularly, and the amount bound to the cellular TCA (trichloroacetic acid) insoluble material, mainly protein and nucleic acids. The $^{14}$C-ethylene labelled derivative is distributed uniformly throughout the medium, since the 3·8% of label found intracellularly is the approximate percentage volume of the cells in the medium. The cyclohexyl labelled nitrosourea attains a higher intracellular concentration, which could be due to breakdown of the agent outside cells and the more efficient uptake of the cyclohexyl moiety, or to the trapping of the moiety intracellularly because of its greater covalent reaction with cell constituents.

Despite the four-fold difference in sensitivity to CCNU, there was no significant difference in the distribution of the compound in the sensitive and resistant tumour lines.

Table III shows the binding of the two labelled derivatives to various macro-

TABLE II.—*Distribution of $^{14}C$ Labelled CCNU*

| Fraction | Sensitive tumour $^{14}$C-ethylene (%) | Sensitive tumour $^{14}$C-cyclohexyl (%) | Resistant tumour $^{14}$C-ethylene (%) | Resistant tumour $^{14}$C-cyclohexyl (%) |
|---|---|---|---|---|
| Total intracellular radioactivity | 3·8 | 14·1 | 6·6 | 14·5 |
| Total extracellular radioactivity | 96·2 | 85·9 | 93·4 | 85·5 |
| TCA insoluble | 15·4 | 29·0 | 14·0 | 30·5 |
| TCA soluble | 84·6 | 71·0 | 86·0 | 69·5 |

Results are expressed as a percentage of total radioactivity. The TCA soluble and insoluble fractions are expressed as a percentage of the total intracellular radioactivity. Results are the averages of 3 determinations.

TABLE III.—*Binding of $^{14}$C-labelled CCNU to Macromolecular Fractions of the TLX5 Lymphoma*

| | Sensitive tumour (d/min/mg dry weight) | | Resistant tumour (d/min/mg dry weight) | |
|---|---|---|---|---|
| Fraction | $^{14}$C-ethylene | $^{14}$C-cyclohexyl | $^{14}$C-ethylene | $^{14}$C-cyclohexyl |
| DNA | 88·5± 8·7 | 54·8± 13·5 | 56·9± 12·4 | 74·3± 12·3 |
| RNA | 227·7±119·6 | 1278·2± 326·3 | 137·9±116·1 | 1311·0± 546·7 |
| Total protein | 410·0± 69·0 | 14768·0±2590·2 | 452·6± 74·5 | 10079·2± 997·0 |
| Cytoplasmic protein | 135·4± 38·2 | 6368·3±1138·1 | 236·6± 98·6 | 5454·8± 451·7 |
| Nuclear protein | 378·3± 86·4 | 15883·6±3342·7 | 513·8±230·9 | 14510·0±1800·1 |

The results obtained with the $^{14}$C-ethylene-labelled compound have been corrected to a specific activity of 12·62 mCi/mmol in order to be directly comparable with the $^{14}$C cyclohexyl labelled derivative. Each result is the mean from 3 separate determinations.

molecules. It is clear that the $^{14}$C-cyclohexyl derivative has a particular affinity for protein, especially the nuclear protein fraction. These results are essentially similar to those of Cheng *et al.* (1972) except that the amount of drug bound to RNA in these experiments is appreciably higher. Analysis of the RNA fraction showed it to contain less than 1% protein and excludes the possibility that the high labelling is an artefact due to contaminating protein. The $^{14}$C-ethylene labelled material, in contrast, showed only a very low degree of binding to any fraction. The results therefore confirm that BCNU reacts predominantly by carbamoylation of lysine residues of proteins following chemical breakdown of the molecule to release cyclohexyl isocyanate (Cheng *et al.*, 1972; Schmall *et al.*, 1973). Carbamoylation of protein would explain the higher radioactivity associated with the nuclear protein fraction compared with cytoplasmic, because of the high concentration of lysine rich protein in the former.

Once again, no difference was found in the amount of drug bound to the various macromolecules of the sensitive and resistant tumour lines.

Although these results show clearly that the majority of the reaction taking place in cells after administration of CCNU involves carbamoylation reactions, this cannot be the sole mechanism of action since the active carbamoylating entity, cyclohexyl isocyanate, while having some properties in common with CCNU is not an effective anti-cancer agent *in vivo* (Oliverio, 1973). However, the high level of reaction with nuclear protein, probably histone, is of interest since it has been claimed that reaction with histone protein is important in the mechanism of action of both alkylating agents and alkyl nitrosamines (Riches and Harrap, 1973; Alonso and Arnold, 1974; Bhattacharya and Schultz, 1974).

The mechanism of action of the antitumour chloroethylnitrosoureas is thus still obscure but it is possible that its action is a complex one involving both inhibition of enzymes and structural proteins by carbamoylation and alkylation of essential macromolecules.

This work was supported by grants to the Chester Beatty Research Institute (Institute of Cancer Research, Royal Cancer Hospital) from the Medical Research Council (Grant No. 973/787/K) and the award of a Medical Research Council Studentship to one of us (J.H.).

### REFERENCES

ALONSO, A. & ARNOLD, H. P. (1974) Stimulation of Amino Acids Incorporation into Rat Liver Nonhistone Chromatin Proteins after Treatment with Diethylnitrosamine. *FEBS Letters*, **41**, 8.

AUDETTE, R. C. S., CONNORS, T. A., MANDEL, H. G., MERAI, K. & ROSS, W. C. J. (1973) Studies on the Mechanism of Action of the Tumour Inhibitory Triazenes. *Biochem. Pharmac.*, **22**, 1855.

BALL, C. R., CONNORS, T. A., DOUBLE, J. A., UJHAZY, V. & WHISSON, M. E. (1966) Comparison of Nitrogen Mustard Sensitive and Resistant Yoshida Sarcomas. *Int. J. Cancer*, **1**, 319.

BHATTACHARYA, R. K. & SCHULTZE, M. O. (1974) Protective Effects of Histones against Drug Induced Alterations of Deoxyribonucleic Acid in Thymus Chromatin. *Biochem. Pharmac.*, **23**, 1519.

Bray, D. A., de Vita, V. T., Adamson, R. H. & Oliverio, V. T. (1971) Effects of 1-(2-chloroethyl)-3-cyclohexyl-1-nitrosourea (CCNU; NSC 79037) and its Degradation Products on Progression of L1210 Cells through the Cell Cycle. *Cancer Chemother. Rep.*, **55**, 215.

Cheng, C. J., Fujimura, S., Grunberger, D. & Weinsten, I. B. (1972) Interaction of 1-(2-chloroethyl)-3-cyclohexyl-1-nitrosourea (NSC 79037) with Nucleic Acids and Proteins *in vivo* and *in vitro*. *Cancer Res.*, **32**, 22.

Gale, G. R. (1965) Effect of 1,3-bis (2-chloroethyl)-1-nitrosourea on *Saccharomyces cerevisiae*. *Proc. Soc. exp. Biol. Med.*, **119**, 1004.

Montgomery, J. A., James, R., McCaleb, G. & Johnston, T. P. (1967) The Modes of Decomposition of 1,3-bis (2-chloroethyl)-1-nitrosourea and Related Compounds. *J. med. Chem.*, **10**, 668.

Oliverio, V. T. (1973) Toxicology and Pharmacology of the Nitrosoureas. *Cancer Chemother. Rep.*, **4** (3), 13.

Pascoe, J. M. & Roberts, J. J. (1974) Interactions between Mammalian Cell DNA and Inorganic Platinum Compounds. II. Interstrand Cross-linking of Isolated and Cellular DNA by Platinum (IV) Compounds. *Biochem. Pharmac.*, **23**, 1345.

Pittillo, R. F., Narkates, A. J. & Burns, J. (1964) Microbiological Evaluation of 1,3-bis(2-chloroethyl)-1-nitrosourea. *Cancer Res.*, **24**, 1222.

Riches, P. G. & Harrap, K. R. (1973) Some Effects of Chlorambucil on the Chromatin of Yoshida Ascites Sarcoma Cells. *Cancer Res.*, **33**, 389.

Schabel, F. M., Johnston, T. P., McCaleb, G. S., Montgomery, J. A., Laster, W. R. & Skipper, H. E. (1963) Experimental Evaluation of Potential Anti-cancer Agents. VIII. Effects of Certain Nitrosoureas on Intracerebral L1210 Leukemia. *Cancer Res.*, **23**, 725.

Schmall, B., Cheng, C. J., Fujimura, S., Gersten, N., Grunberger, D. & Weinstein, I. B. (1973) Modification of Proteins by 1-(2-chloroethyl)-3-cyclohexyl-1-nitrosourea (NSC 79037) *in vitro*. *Cancer Res.*, **33**, 1921.

Schmid, F. A. & Hutchinson, D. J. (1972) Collateral Sensitivity of Resistant Lines of Mouse Leukemias L1210 and L5178Y. *Cancer Res.*, **32**, 808.

Selawry, O. S. & Hansen, H. H. (1972) Superiority of CCNU (1-(2-chloroethyl)-3-cyclohexyl-1-nitrosourea; NSC 79037) over BCNU (1,3-bis(2-chloroethyl)-1-nitrosourea; NSC 409962) in Treatment of advanced Hodgkin's Disease. *Proc. Am. Ass. Cancer Res.*, **13**, 46.

Shirakawa, S. & Frei, E. (1970) Comparative Effects of the Anti-tumor Agents 5(dimethyltriazeno)imidazole-4-carboxamide and 1,3-bis(2-chloroethyl)-1-nitrosourea on cell cycle of L1210 Leukemia cells *in vivo*. *Cancer Res.*, **30**, 2173.

Wheeler, G. P. & Bowden, B. J. (1965) Some Effects of 1,3-bis(2-chloroethyl)-1-nitrosourea Upon the Synthesis of Protein and Nucleic Acids *in vivo* and *in vitro*. *Cancer Res.*, **25**, 1770.

Wheeler, G. P. & Chumley, S. (1967) Alkylating Activity of 1,3-bis(2-chloroethyl)-1-nitrosourea and Related Compounds. *J. med. Chem.*, **10**, 259.

Young, R. C. (1969) Changes in the DNA Synthetic Phase of the Cell Cycle of Leukemia L1210 Induced by the Chemotherapeutic Agents, 1,3-bis(2-chloroethyl)-1-nitrosourea (BCNU) and Cyclophosphamide (CTX). *Proc. Am. Ass. Cancer Res.*, **10**, 102.